# Perinatal depressive symptoms in Portuguese new fathers and mothers during COVID-19 pandemic

**DOI:** 10.1192/j.eurpsy.2023.1656

**Published:** 2023-07-19

**Authors:** D. Pereira, A. Macedo, C. Cabaços, B. Wildenberg, N. Madeira, A. T. Pereira

**Affiliations:** 1 Department of Psychological Medicine, Faculty of Medicine, University of Coimbra; 2 Department of Psychiatry, Centro Hospitalar e Universitário de Coimbra, Coimbra, Portugal

## Abstract

**Introduction:**

Postpartum depression, refers to depressive symptoms within a 12-month period after the birth of an infant in a new father or mother. It’s have been a growing mental health concern, as it is one of the leading causes of poor familial and infant health outcomes. Despite the growing attention being given to fathers’ depression, including in Portugal, prevalence data and its possible correlates are still scarce compared to depression in mothers, especially during the COVID-19 pandemic.

**Objectives:**

To explore and compare levels of depressive symptomatology and to analyze potential correlates for postpartum depression in Portuguese new mothers and fathers during the COVID-19 pandemic.

**Methods:**

153 men and 187 women (mean age: 36.61± 4.99 vs. 32.98 ±5.00 years, respectively) were recruited in the perinatal period (7.29±3.22 vs 8.58± 0.97 months post-partum) and answered to an online survey that included questions related to sociodemographic and psychosocial variables and validated questionnaires: Perinatal Depression Screening Scale (PDSS), Perseverative Thinking Questionnaire (for Repetitive Negative Thinking/RNT) and Dysfunctional Beliefs Towards Motherhood/Fatherhood Scale (DBTM/F). Statistical analysis was performed using IBM Statistical Package for the Social Sciences (SPSS Version 26 for Mac).

**Results:**

New-mothers had significantly higher levels of depressive symptoms than new-fathers (41.89±16.94 VS. 33.95±14.99, p<.001). Based on the PDSS’ cutoff point the prevalence of clinically relevant depressive symptoms in male and female progenitors was 21.6% and 39.6%, respectively (p<.001).

DBTF were significantly higher compared to DBTM (p<.05). Male and female progenitors did not differ regarding levels of RNT.

DBT-M/F (r».40) and RNT (r>.55) significantly and positively correlated with PDSS scores.

In both genders, DBT-M/F and RNT significantly (p<.01) predicted PDSS scores explaining 33.8% (Beta: DBT=.136, p=.050; RNT=.538, p<.01) of its variance in fathers and 50.4% in mothers (Beta: DBT=.218, p=.001; RNT=.565, p<.01).

**Conclusions:**

During the COVID-19 pandemic, Portuguese recent mothers had higher levels of depressive symptoms when compared to recent fathers. However, in both new mothers and fathers, depressive symptoms’ prevalence and severity were higher than the figures found in samples of new parents outside of the pandemic period and of samples from the general population. Addressing DBT-M/F, as well as RNT, in recent parents, could be relevant in preventing/improving their depressive symptoms.

**Disclosure of Interest:**

None Declared

